# A Single-Step Surface Modification of Electrospun Silica Nanofibers Using a Silica Binding Protein Fused with an RGD Motif for Enhanced PC12 Cell Growth and Differentiation

**DOI:** 10.3390/ma11060927

**Published:** 2018-05-30

**Authors:** Wen Shuo Chen, Ling Yu Guo, Amien Mohamed Masroujeh, Anna Morgan Augustine, Cheng Kang Tsai, Ting Yu Chin, Yui Whei Chen-Yang, Mong-Lin Yang

**Affiliations:** 1 Department of Chemistry, Center for Nanotechnology, Center for Biomedical Technology, Chung Yuan Christian University, Chung Li 32023, Taiwan; allanson92@yahoo.com.tw (W.S.C.); sunny_day80917@yahoo.com.tw (L.Y.G.); chengkang20071994@gmail.com (C.K.T.); 2 Department of Science, Concordia University Saint Paul, Saint Paul, MN 55104, USA; amasroujeh@gmail.com (A.M.M.); aaugustine@atsu.edu (A.M.A.); 3 Department of Bioscience Technology, Chung Yuan Christian University, Chung Li, 32023, Taiwan; tychin@cycu.edu.tw

**Keywords:** electrospun silica nanofibers, silica binding protein, RGD, neuronal tissue engineering

## Abstract

In this study, a previously known high-affinity silica binding protein (SB) was genetically engineered to fuse with an integrin-binding peptide (RGD) to create a recombinant protein (SB-RGD). SB-RGD was successfully expressed in *Escherichia coli* and purified using silica beads through a simple and fast centrifugation method. A further functionality assay showed that SB-RGD bound to the silica surface with an extremely high affinity that required 2 M MgCl_2_ for elution. Through a single-step incubation, the purified SB-RGD proteins were noncovalently coated onto an electrospun silica nanofiber (SNF) substrate to fabricate the SNF-SB-RGD substrate. SNF-SB-RGD was characterized by a combination of scanning electron microscopy (SEM), Fourier transform infrared (FTIR) spectroscopy, and immunostaining fluorescence microscopy. As PC12 cells were seeded onto the SNF-SB-RGD surface, significantly higher cell viability and longer neurite extensions were observed when compared to those on the control surfaces. These results indicated that SB-RGD could serve as a noncovalent coating biologic to support and promote neuron growth and differentiation on silica-based substrates for neuronal tissue engineering. It also provides proof of concept for the possibility to genetically engineer protein-based signaling molecules to noncovalently modify silica-based substrates as bioinspired material.

## 1. Introduction

Nanoscale biomaterials is a rapidly expanding field of research that has seen great application success in areas such as cancer therapy [1,2], gene delivery [3,4], antimicrobial resistance [5,6], and tissue engineering [7,8]. The neuronal tissue engineering field has especially focused on producing three-dimensional, bioactive, and biodegradable nanoscaffolds that mimic the extracellular matrix (ECM) [9] as a promising approach for nerve repair and nervous system regeneration [10,11]. Amongst the more popular studied scaffolds, certain limiting factors that hinder their applications have been reported. For example, natural materials, such as collagen and laminin, could experience inconsistency from batch to batch, while the production of synthetic organic polymers, such as polycaprolactone and poly(lactic-co-glycolic acid), would require the use of toxic organic solvents for dissolution [12]. Therefore, more recent explorations have identified electrospun silica nanofibers as a three-dimensional, nontoxic, biocompatible, bioactive, and biodegradable inorganic-based alternative for neuronal tissue engineering [13,14,15].

In studying neuronal tissue engineering, an abundance of literature has highlighted the importance of providing surface contact guidance cues in the form of ECM proteins for developing neurons [16,17]. Within the ECM proteins, such as fibronectin, collagen, vitronectin, and laminin [18,19], an Arg-Gly-Asp (RGD) motif was found to improve cell adhesion to material surfaces [20] and is widely used for tissue engineering to promote cell growth [20,21,22]. However, the procedures for immobilizing RGD-containing peptides onto material surfaces usually demand tedious cross-linking processes with limited yield [23,24]. Moreover, the chemical modification processes in non-aqueous solution could reduce the biological function of the adhesion molecules [25,26]. Therefore, having an immobilization method that is non-damaging and effective for the binding of an RGD-containing protein onto material surfaces is desired. Some studies have found that genetically engineered fusion proteins, such as RGD-CBD (cellulose binding domain) [20], RGD-SBM (starch binding module) [27], and RGD-PhaP (polyhydroxyalkanoate granule binding protein) [21,28], could easily be used to surface modify their counterpart materials with high affinity. In addition, an RGD-phage coating has also been shown to be an alternative noncovalent surface modification for material surfaces [29]. However, so far, no study has applied this idea of utilizing an efficient, noncovalent surface modification for grafting RGD onto electrospun silica nanofibers (SNFs) in neuronal tissue engineering.

We have previously demonstrated a multi-step chemical reaction process to modify the surface of a silica nanofiber for assisting cell attachment and enhancing neuronal cell growth and differentiation [30]. To explore the possibility of grafting RGD onto silica nanofibers without introducing any cross-linking agents, we identified a published silica-binding protein (SB) [31] that could be used for one-step anchoring of RGD onto silica surfaces. This present work shows the successful design and engineering of a novel fusion protein SB-RGD, which contains the SB protein for mediating silica surface binding of the SNF substrate and the RGD motif for allowing integrin-receptor-dependent cell adherence. In addition, a simple and rapid purification method leveraging the silica binding ability of the fusion protein is described. The purified SB-RGD was functionally shown to bind to the SNF surfaces with high affinity. The heat-denatured SB-RGD was found to have limited binding ability to the SNF surface, affirming that the purified SB-RGD retained proper folding to assist noncovalent binding to the SNF surface. Last, the as-prepared RGD surface-functionalized SNF (SNF-SB-RGD) was proved to enhance the adhesion of PC12 cells for its survival and sustained neurite outgrowth for its differentiation. We believe that this work presents a new surface modification approach that requires only single-step incubation and could easily be genetically engineered to present various other protein-based signaling molecules for a more versatile neuronal tissue engineering scaffold.

## 2. Materials and Methods

### 2.1. Materials

Tetraethyl orthosilicate (TEOS), the silica precursor, was purchased from Acros Organics (Thermo Fisher Scientific, Waltham, MA, USA). Polyvinyl pyrrolidone (PVP, Mw = 1,300,000 g/mol) and phosphate buffered saline (PBS) were purchased from Sigma-Aldrich Co. (St. Louis, MO, USA).

### 2.2. Preparation and Characterization of SNF Substrates

The SNF was prepared on a coverslip by the electrospinning (ES) method reported previously [14,15]. A mixture of 1.9 g of TEOS, 0.04 g of formic acid, 3.15 g of ethanol, and 2.0 g of water was mixed with 0.9 g of PVP then continuously stirred for 1 h to form the ES solution. The silica/PVP composite nanofibers were firstly prepared from the ES solution by the ES technique at a flow rate of 0.9 mL/h using a 24-G plastic syringe with a stainless steel needle. The voltage applied was 16 kV, and the distance from a 12 × 12 mm^2^ coverslip placed on a flat aluminum plate collector was 10 cm. The silica/PVP composite nanofibers collected on the coverslip were finally calcined for 3 h at 450 °C to remove the PVP and the solvent residues to form SNF.

The morphologies of all the as-prepared silica nanofibers were studied with an S-3500N field emission SEM (Hitachi, Tokyo, Japan). The diameters of the silica nanofibers prepared were measured from the SEM images with Image J analysis software (50 fibers for each sample, n = 50). FTIR (BIORad-FTS-7, Perkin Elmer, Waltham, MA, USA) spectra of the SNF and SNF-SB-RGD substrates were also measured over 4000–400 cm^−1^ at a resolution of 2 cm^−1^ to identify the functional groups of the silica nanofibers.

### 2.3. Plasmid Construction and the Expression and Purification of the SB-RGD Protein

For the construction of the plasmid pET-SB-RGD, we first digested the pET21b plasmid (Novagen, Madison, WI, USA) with NheI and XhoI. The silica binding protein SB (ribosomal protein L2; rplB) sequence (1082440-1083261 nt, GenBank Accession No. NZ_CP027060), linker (5′-GGAGGTGGAGGTTCCTCATCCTCATCC-3′), and RGD sequence (5′-GGACGGGGCGATTCC-3′) with the NheI restriction site at the 5′ end and the XhoI restriction site at the 3′ end was chemically synthesized by Genomics, New Taipei City, Taiwan. The SB-RGD sequence was then subcloned into the NheI and XhoI sites of pET21b to obtain the plasmid pET-SB-RGD.

Recombinant *Escherichia coli* Rosetta (DE3) pLysS (Novagen) was used as the host strain to produce the fusion protein SB-RGD. The recombinants were cultivated overnight in 200 mL Luria Bertani medium with 100 μg/mL ampicillin in the presence of 0.1 mM isopropyl b-D-thiogalactopyranoside (IPTG) (Sigma-Aldrich). The cultivated cells were centrifuged at 8000g for 8 min and lysed using lysozyme (Thermo Fisher Scientific) with further centrifugation at 15,000× *g* for 20 min to remove cell debris. The recovered whole-cell lysate was incubated with silica particles (approximately 20 mg) for 30 min at room temperature. The particles were centrifuged at 15,000× *g* for 10 min and washed twice with 5 mL of wash buffer of 25 mM Tris buffer containing 0.5 M NaCl. Next, the particles were further suspended in 5 mL of 25 mM Tris buffer containing 2 M MgCl_2_ for elution. After a 20-min incubation, the suspension was centrifuged at 15,000× *g* for 10 min. The obtained supernatant containing the purified SB-RGD proteins was collected and dialyzed against 25 mM Tris buffer containing 0.5% Tween 20 to remove the MgCl_2_. The protein concentration of samples was determined by the protein assay kit (Bio-Rad Laboratories Inc., Hercules, CA, USA). About 15 μg of lysate from each sample was loaded and separated by SDS-PAGE on 12.5% polyacrylamide gels under reducing conditions.

### 2.4. Dissociation Testing of SB-RGD from Silica Particles

The whole-cell lysate obtained as previously described in Section 2.3 was incubated with silica particles (approximately 20 mg) for 30 mins. Then, the SB-RGD-bound silica particles were separated out through centrifugation at 15,000× *g* for 10 min and then incubated in the following solutions at room temperature for 30 mins: (1) 25 mM Tris buffer containing 2M NaCl; and (2) 25 mM Tris buffer containing 2 M MgCl_2._ After incubation, the silica particles were collected by centrifugation at 15,000× *g* for 10 min and washed three times with 5 mL of 25 mM Tris buffer. Finally, each sample was centrifuged at 15,000× *g* for 10 min, boiled at 95 °C for 15 min, and analyzed by SDS–PAGE.

### 2.5. Preparation of SNF-SB-RGD and the SNF Coated with Heat-Denatured SB-RGD (SNF-SB-RGD∆)

**Figure 1:**
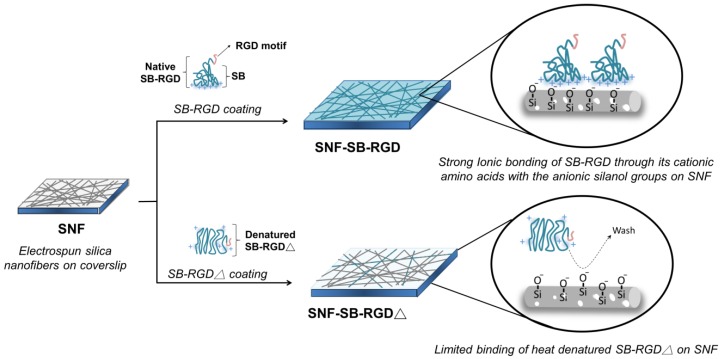
Schematic of the various prepared substrates. SNF: the uncoated mesoporous electrospun silica nanofiber on a coverslip. SNF-SB-RGD: the SNF substrate coated with purified native SB-RGD fusion protein that strongly bound to SNF surface through ionic bonding. SNF-SB-RGD∆: SNF substrate coated with SB-RGD∆, which yielded limited binding after PBS washing due to denatured conformation.

The various electrospun silica nanofiber substrates, SNF, SNF-SB-RGD, and SNF-SB-RGD∆, utilized in this work are illustrated in Figure 1. To prepare SNF-SB-RGD, 0.01 mg/mL purified SB-RGD was incubated with SNF for 1 h then washed three times with PBS to remove any unbound proteins. For the fabrication of SNF-SB-RGD∆, purified SB-RGD was first heated at 95 °C for 15 min to obtain SB-RGD∆, then 0.01 mg/mL SB-RGD∆ was incubated with SNF for 1 h followed by three washes with PBS.

### 2.6. Examining the Immobilization of Fusion Protein on SNF-SB-RGD and SNF-SB-RGD∆

To visually examine the immobilization of fusion protein to the silica material, the substrates were first blocked using 10% goat serum (Thermo Fisher Scientific). Then, the samples were incubated with mouse anti-his tag primary antibody (diluted at 1:500, Merck & Co. Inc., Kenilworth, NJ, USA). After washing with PBS, the samples were immersed with DyLight 488 conjugated donkey anti-mouse (1:250 dilution; Jackson ImmunoResearch, West Grove, PA, USA) antibody for 1.5 h at 37 °C and washed again with PBS before imaging. Ten images at different positions were acquired under fluorescence microscopy (Nikon, Shinagawa, Tokyo, Japan). For each sample, 10 pairs of regions of interest (ROI) on each fluorescence image, including those from fibers and those from the corresponding backgrounds, were randomly selected utilizing the ImageJ software. The average signal intensity of each region was measured by tracing 50 pixels either on the background or along the fiber. The ratio was then calculated with the average signal intensity of the ROI obtained from the fiber over that from the corresponding background.

### 2.7. In Vitro Culture of PC12 Cells

Rat PC12 cells (American Type Culture Collection, Manassas, VA, USA), previously used to explore scaffold for nerve regeneration [15,32], were cultured in high-glucose DMEM supplemented with 5% heat-inactivated fetal bovine serum, 10% horse serum, and 1% penicillin/streptomycin at 37 °C in a humid atmosphere with 5% CO_2_ [33]. For seeding of PC12 cells onto the various silica nanofiber substrates, the live cells were counted with a trypan blue exclusion assay in a hemocytometer. Then, PC12 cells with a density of 6.9 × 10^3^ cells/ cm^2^ were seeded onto the various silica nanofiber substrates sterilized overnight under UV radiation. For neuronal differentiation, the cells after seeding were cultured in DMEM supplemented with 1% heat-inactivated fetal bovine serum, 2% horse serum, and 1% penicillin/streptomycin supplemented with 100 ng/mL nerve growth factor (NGF) (Corning, NY, USA). For cell maintenance, the medium was replenished every 3 days.

### 2.8. Cell Viability Assay

PC12 cell viability was determined using a Live/Dead assay kit (Life Technologies, Waltham, MA, USA). The PC12 cells were first seeded on the as-prepared SNF substrates then assayed after 72 h of cultivation [15,34]. Calcein AM and Ethidium Homodimer-1 dyes diluted in PBS to a final concentration of 1 μM were added to the cultivated cells for 10 min. Then, 10 images at different positions (n = 10) were taken for each sample utilizing the fluorescence microscope (Nikon). The cells stained with Calcein AM, a cell-permeant dye, were counted as live cells, whereas those stained with EthD-1, a membrane-impermeable DNA-binding dye, were counted as dead cells. Based on the ratio of the number of live cells to that of the total cells, the percentage of viable cells for each sample was calculated.

### 2.9. Immunocytochemistry Staining

The fluorescence immunocytochemistry conducted on the PC12 cells was similar to the procedure previously reported [35,36]. Briefly, the cells were fixed with 4% paraformaldehyde (PFA, Merck & Co. Inc.) for 30 min, then blocked with 10% goat serum and 0.3% Triton X-100 in PBS for 2 h. After overnight incubation at 4 °C with rabbit anti-microtubule-associated protein 2 (MAP2) antibody (1:500 dilution; Merck & Co. Inc.), the cells were washed and further incubated with secondary antibodies, DyLight 488 conjugated donkey anti-rabbit (1:250 dilution; Jackson ImmunoResearch) antibody, for 1.5 h at 37 °C. Twenty-three fluorescence images from various fields were acquired using fluorescence microscopy (Nikon) for each sample using the 20× objective lens. The neurite lengths indicated by the MAP2 staining were analyzed with the Neuron J software (National Institutes of Health, Bethesda, MD, USA) [37] plugin of Image J [38].

### 2.10. Cell Morphology Study

The morphologies of the PC12 cells on the SNF substrates were examined with the SEM. On the 5th day after seeding, the substrates with cells were collected, then fixed with formaldehyde and sequentially dehydrated via an increasing concentration gradient of alcohol. Finally, the substrates were dried at room temperature and coated with gold for observation by the SEM.

### 2.11. Statistical Analysis

All of the data are presented as means ± standard error of mean. The difference was determined by one-way analysis of variance (ANOVA) or the student’s *t*-test. The statistical significance was taken at *p* < 0.05.

## 3. Results and Discussion

### 3.1. Genetic Engineering and Expression of the Recombinant SB-RGD Fusion Protein.

**Figure 2:**
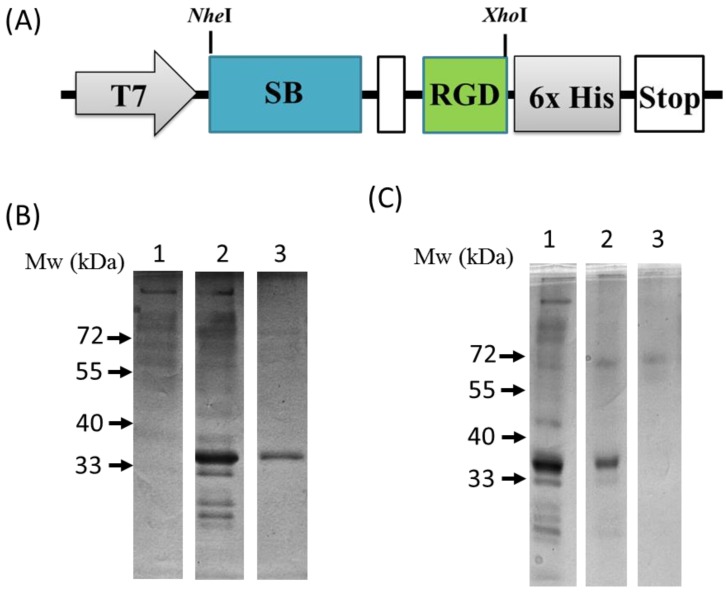
(**A**) Schematic representation of the cloning site for genetic engineering of the fusion protein SB-RGD. (**B**) The SDS-PAGE result of the expressed and purified fusion proteins. Lane 1: pre-induced whole-cell lysate (negative control); lane 2: soluble fractions (crude proteins) of induced SB-RGD; lane 3: the purified SB-RGD protein. (**C**) The SDS-PAGE result of the SB-RGD dissociation assay. Lane 1: the induced whole-cell lysate; lane 2: the silica particles immersed in the induced whole-cell lysate, washed by incubation with 25 mM Tris buffer (pH 8.0) containing 2 M NaCl for 30 min, and finally boiled at 95 °C for 15 min with SDS sample buffer before being loaded into SDS-PAGE for analysis. Lane 3: sample treated exactly the same as lane 2 except that the wash was conducted with 25 mM Tris buffer (pH 8.0) containing 2 M MgCl_2_.

As seen in Figure 2A, a recombinant plasmid (pET-SB-RGD) was genetically engineered to have the SB protein (depicted as a blue box) fused in-frame through a linker sequence (depicted as a white box) to the RGD motif (depicted as a green box) followed by six histidines (depicted as a gray box) for immune-detection that ended with a stop codon. The sequencing-verified recombinant plasmid was then transformed into a host strain *Escherichia coli* Rosetta (DE3) pLysS for expression. Successful production of the SB-RGD fusion proteins was achieved through IPTG induction. As seen in the SDS-PAGE analysis presented in Figure 2B, lane 2, after IPTG induction, the whole-cell lysate yielded a prominent SB-RGD recombinant protein band at 35 kD, which was not present in the preinduction sample lane (Figure 2B, lane 1).

### 3.2. Purification and Functional Testing of SB-RGD

To functionally test the silica binding ability of the fusion protein, as well as leveraging the silica binding ability for a simple purification method, we incubated the induced cell lysate with silica particles. After incubation was done to allow for binding, the silica particles were washed with either 25 mM Tris buffer with 2 M NaCl, or 25 mM Tris buffer with 2 M MgCl_2_ to test the binding affinity. The washed silica particles were then cooked with SDS sample buffer and loaded into SDS-PAGE for protein analysis. Figure 2C, lane 1, shows the whole-cell lysate that was used to coat the silica particles. Figure 2C, lane 2, demonstrates that, after a high-salt wash with 2 M NaCl, most proteins were gone while the SB-RGD protein stayed bound to the silica particle; this is evident in the presence of the single SB-RGD band in lane 2. This result functionally shows that the SB-RGD protein can bind tightly through high affinity to the silica surface as predicted. Further testing was done to investigate the condition for dissociating the SB-RGD from the silica particle. The result shows that divalent cations Mg^2+^ at the concentration of 2 M are effective at releasing the SB-RGD protein, which is evident through the lack of an SB-RGD protein band in Figure 2C, lane 3. These results of SB silica binding behavior are consistent with the previous study [39] and confirm the strong silica binding ability of the expressed SB-RGD protein.

After defining the dissociation condition of the SB-RGD protein from the silica particles, we were able to establish a simple purification method that does not require conventional chromatography but only simple centrifugation for rapid separation. Specifically, we start by incubating the induced lysate with silica particles, followed by washes done through incubation and separation done through centrifugation. The elution was performed by incubating the washed particles in 2 M MgCl_2_. Last, after the silica particles were pelleted by centrifugation, the resulting eluent was dialyzed against neutral 25 mM Tris buffer to obtain purified SB-RGD. As seen in Figure 2B, lane 3, when purified SB-RGD was analyzed by SDS-PAGE, the result shows a clean band at approximately 35 KDa.

### 3.3. Preparation and Characterization of SNF-SB-RGD

To obtain the desired SNFs with noncovalently modified RGD motifs, SNF was first prepared as previously published [30]. With the SB-RGD now proven to have a high binding affinity to the silica surface, the production of SNF-SB-RGD was done simply through a one-step incubation of 0.01 mg/mL of the fusion protein with the SNF substrate for 1 h.

**Figure 3:**
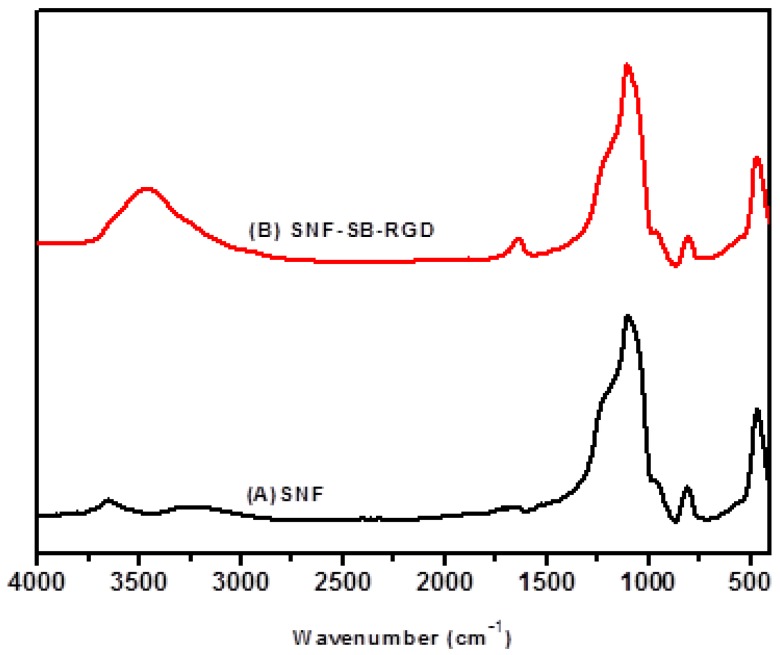
FTIR spectra of (**A**) SNF and (**B**) SNF-SB-RGD.

**Figure 4:**
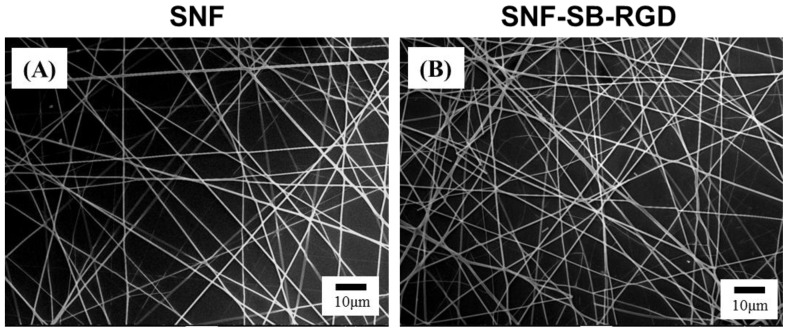
SEM images of (**A**) SNF and (**B**) SNF-SB-RGD. The scale bar represents 10 μm.

FTIR analysis proved the successful ionic bonding of SB-RGD onto the silica surfaces of the SNF-SB-RGD substrate. As seen in Figure 3B, the characteristic band of peptide bonds (1700–1600 cm^−1^ for the C=O stretching vibration of amide I and 3500–3300 cm^−1^ for the N-H stretching vibration) [40,41] indicates that the peptide bond of SB-RGD was successfully introduced onto the surface of ionically bonded silica nanofibers (SNF-SB-RGD). In addition, the characterization done through SEM micrographs showed that the fiber diameters of the SNF (612 ± 57.0 nm) and SNF-SB-RGD (616 ± 83.7 nm) substrates did not change significantly (Figure 4, n = 50, *t*-test, *p* > 0.1). 

### 3.4. Binding of SB-RGD to the SNF Substrate’s Surface

**Figure 5:**
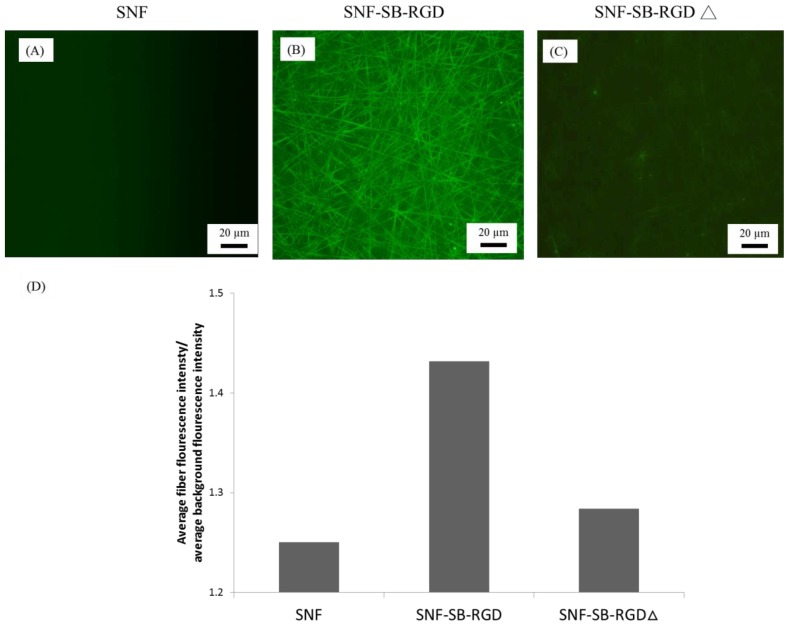
Fluorescence images of the SNF substrates coated with SB-RGD or SB-RGD∆ (95 °C, 15 min) immunostained with the anti-his tag antibody. (**A**) SNF, (**B**) SNF-SB-RGD, and (**C**) SNF-SB-RGD∆. (**D**) Fluorescence analysis of the SNF substrates (n = 10). The scale bars represent 20 µm.

To verify that the noncovalent modification of SB-RGD to the silica surface of SNF requires a functional structure moiety of SB, we compared the binding of SB-RGD versus SB-RGD∆ (SB-RGD denatured through heating at 95 °C for 15 min) to SNF. Immunocytochemistry staining using an anti-his tag antibody and imaged under fluorescent microscopy was employed to detect the amount of SB-RGD bound to the SNF surfaces. As shown in Figure 5, under the same exposure time, the fluorescent microscopy image of SNF (Figure 5A) reveals a baseline fluorescent signal, while SNF-SB-RGD (Figure 5B) gave a much stronger and more visible signal, and SNF-SB-RGD∆ (Figure 5C) had a dim and limited fluorescent signal. To quantify this data, we used the image analysis software Image J [38] to obtain the average signal intensity from 10 randomly selected regions of 50 pixels on both the electrospun fiber and the background. The ratio of the average intensities gathered from the fiber over the background is reported on the y-axis of Figure 5D. This data confirms that the purified SB-RGD retained its silica-binding functionality, as such binding ability can be disrupted through heat denaturation. This also affirms that, to obtain a strong attachment through the ionic binding with SNF, a proper folding of the coating biologic is required.

### 3.5. Effect of Fusion-Protein-Modified SNF on Cell Viability

**Figure 6:**
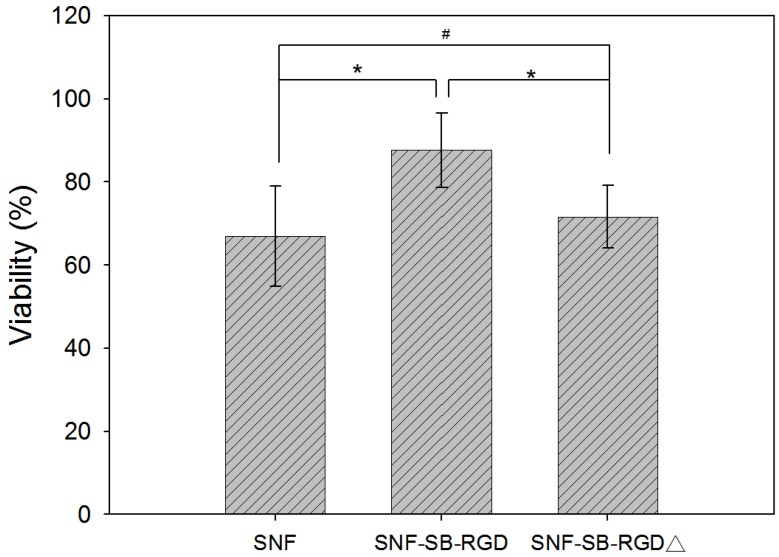
Cell viability comparison between PC12 cells cultured on SNF, SNF-SB-RGD, and SNF-SB-RGD∆ substrates. Assessments were done using the LIVE/DEAD® stain with Ethidium Homodimer-1 and Calcein AM 72 hours after seeding. n = 10 *t*-test, * *p* <0.05, # *p* > 0.05.

Next, a LIVE/DEAD assay was performed to test the biocompatibility of the different substrates. The result shows that although the cell viability on SNF-SB-RGD is lower than that on the conventional control (as shown in Supplementary Materials Figure S1), it was significantly higher than the unmodified SNF (66.87 ± 12.06%) and the denatured fusion-protein-coated SNF, SNF-SB-RGD∆ (71.63 ± 7.47%)(n = 10 *t*-test, * *p* <0.05 and ^#^ *p* >0.05) (Figure 6). This result reaffirms that PC12 cells could not easily adhere and grow onto the uncoated surface of SNF, which is consistent with a previous report [42]. However, on the surface successfully modified by SB-RGD (SNF-SB-RGD), significant cell adherence and therefore growth and viability were observed. When SB-RGD is denatured through heating (SNF-SB-RGD∆), the surface binding ability is disrupted due to the change of conformation, and the cell viability rate is similar to that of uncoated SNF (SNF). This result shows the biocompatibility of the SNF-SB-RGD substrate and demonstrates the importance of a functional fusion protein with proper folding for successful surface modification in sustaining cell growth.

### 3.6. Effect of the Various Substrates on the Neurite Extension of PC12 Cells

**Figure 7:**
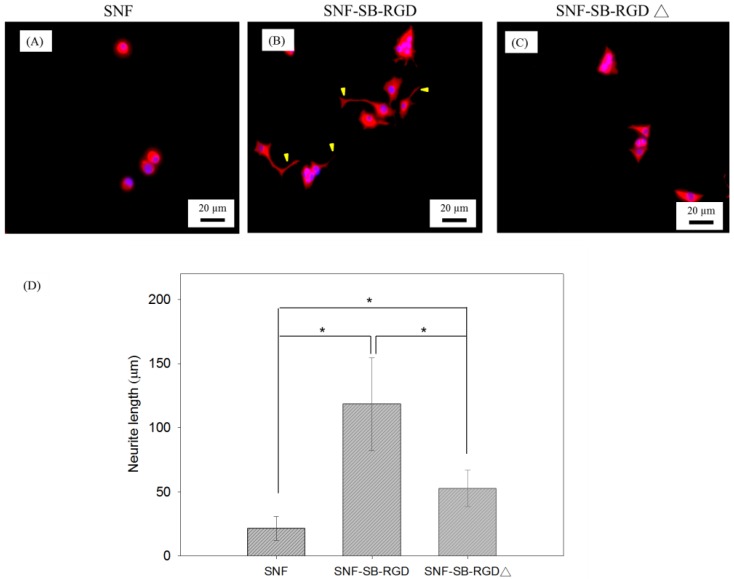
Fluorescence microscopy image of PC12 cell differentiation on (**A**) SNF, (**B**) SNF-SB-RGD, and (**C**) SNF-SB-RGD∆ substrates at Day 5. Neurite (indicated with arrowheads) was immunostained with MAP2 (red). (**D**) Bar graph comparing the neurite length of PC12 cells cultured on the as-prepared SNF substrates. The scale bars represent 20 μm. (n = 23, *t*-test, * *p* < 0.05).

To test the effect of SNF-SB-RGD on sustaining cell differentiation, NGF-induced PC12 cell differentiation on SNF, SNF-SB-RGD, and SNF-SB-RGD∆ substrates was assessed via immunocytochemical staining against MAP2. Figure 7 shows fluorescent microscopy images of PC12 neurite outgrowth on the three substrates. The images show that the SNF-SB-RGD substrate can more effectively promote and sustain neurite extension throughout the 5 days observed (Figure 7B) compared to SNF (Figure 7A) and SNF-SB-RGD∆ (Figure 7C). Neurite length was determined via image analysis software using methods previously established [37]. As summarized in Figure 7D, cells seeded on SNF exhibited minimal neurite extension (21.42 ± 9.35 μm) throughout the time observed, while cells on ionically bonded SNF-SB-RGD exhibited longer neurite lengths (118.32 ± 36.39 μm). Not surprisingly, cells on the SNF-SB-RGD∆ substrate failed to sustain and exhibited significant neurite extension (52.67 ± 14.26 μm), indicating that SNF-SB-RGD can sustain neurite outgrowth longer than SNF-SB-RGD∆ due to the binding ability of SB-RGD onto SNF.

**Figure 8:**
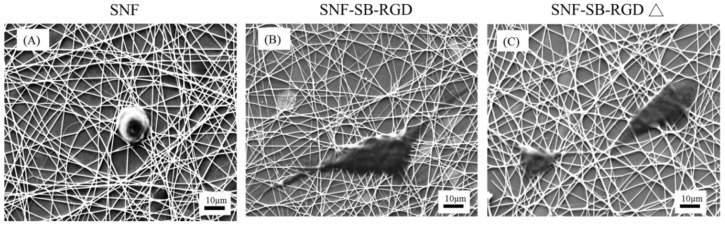
SEM images of PC12 cell differentiation on (**A**) SNF, (**B**) SNF-SB-RGD, (**C**) SNF-SB-RGD∆ substrates at Day 5. The scale bars represent 10 μm.

Last, to visualize the structure and morphology of the cell–scaffold interaction on our substrates, SEM images were analyzed for closer inspection of the cell morphology on all substrates. As seen in Figure 8, SNF-SB-RGD (Figure 8B) is able to support cell growth with enhanced neurite outgrowth compared to uncoated SNF (Figure 8A) or SNF-SB-RGD∆ (Figure 8C). For the SNF-SB-RGD∆ with limited surface-bound RGD peptides, cells appear round and less defined, implying limited adhesion without differentiation. In contrast, for the SNF-SB-RGD which has a significant amount of RGD peptides bound, cells grown on the surface appeared flatter (indicating cell adhesion) with pronounced protrusions (indicating cell differentiation). These results showed that the SNF-SB-RGD substrate was able to promote cell attachment to the substrate surface and encourage neurites interaction with the three-dimensional structure as seen by it weaving in and out of the silica nanofibers.

The success of this work presents an alternative way to modify the surfaces of inorganic tissue engineering scaffolds through recombinant proteins that have a high affinity for the silica substrate. It also opens the possibility to create an array of silica-binding biologics that present various stimulating signals (such as RGD, IKVAV, YIGSR, etc.) for cell growth or differentiation guidance on a silica nanofiber scaffold for tissue engineering. Furthermore, such an array would also have the potential to be used in silica nanoparticle modification for drug delivery related to cancer therapy [43].

## 4. Conclusions

In this work, a recombinant fusion protein, SB-RGD, was genetically designed to allow for easy purification as well as a simple one-step surface modification of SNF. This new convenient and effective approach to a coating process avoids the complex and potentially damaging chemical reactions that are typically employed for covalent modification. Our results showed that SB-RGD possessed a strong affinity for the silica surface to serve as an anchoring molecule and as a motif for cell adhesion. Both the cell viability assay and neurite outgrowth analysis of PC12 cells found SNF-SB-RGD to be significantly better than the uncoated control, SNF, and the heat-denatured control, SNF-SB-RGD∆. Overall, we present a novel effective method for surface modification of silica nanofibers for neuronal tissue engineering.

## Supplementary Materials

The following are available online at http://www.mdpi.com/1996-1944/11/6/927/s1, Figure S1: Cell viability comparison between PC12 cells cultured on SNF-SB-RGD and conventional control, laminin-coated tissue culture polystyrene (TCPS-laminin), which served as the positive control. Cell viability on SNF-SB-RGD was 87.63 ± 9.02% and on TCPS-laminin 90 ± 7.68%. Assessments were done using LIVE/DEAD® stain with Ethidium Homodimer-1 and Calcein AM 72 hours after seeding. n = 10 *t*-test, # *p* > 0.05.5.
