# Supplementary material for: A Single-Step Surface Modification of Electrospun Silica Nanofibers Using a Silica Binding Protein Fused with an RGD Motif for Enhanced PC12 Cell Growth and Differentiation
Source: Materials (Basel). 2018 May 30;11(6):927. doi: 10.3390/ma11060927 (PMC6024934; doi:10.3390/ma11060927)
Supplement: Supplementary file 1 [file materials-11-00927-s001.pdf]

Supplementary Information

A single-step surface modification of electrospun silica nanofibers using silica binding protein fused with RGD motif for enhanced PC12 cells growth and differentiation

Wen Shuo Chen,[1] Ling Yu Guo,[1] Amien Mohamed Masroujeh,[2] Anna Morgan Augustine,[2] Cheng Kang Tsai,[1] Ting Yu Chin,[3] Yui Whei Chen-Yang [1,*] and Mong-Lin Yang [2,*]

1 Department of Chemistry, Center for Nanotechnology, Center for Biomedical Technology, Chung Yuan Christian University, Chung Li, 32023, Taiwan; allanson92@yahoo.com.tw (W.S.C.); sunny_day80917@yahoo.com.tw (L.Y.G.); chengkang20071994@gmail.com (C.K.T.)

2 Department of Science, Concordia University Saint Paul, Saint Paul, Minnesota, 55104,USA; amasroujeh@gmail.com (A.M.M.); aaugustine@atsu.edu (A.M.A.)

3 Department of Bioscience Technology, Chung Yuan Christian University, Chung Li, 32023, Taiwan; tychin@cycu.edu.tw (T.Y.C.)

* Correspondence: myang2@csp.edu (M.-L.Y.); yuiwhei@cycu.edu.tw (Y.W.C-Y.); Tel.: +1-651-6418220 (M.-L.Y.); Tel.: +866-3-2653317 (Y.W.C-Y.)

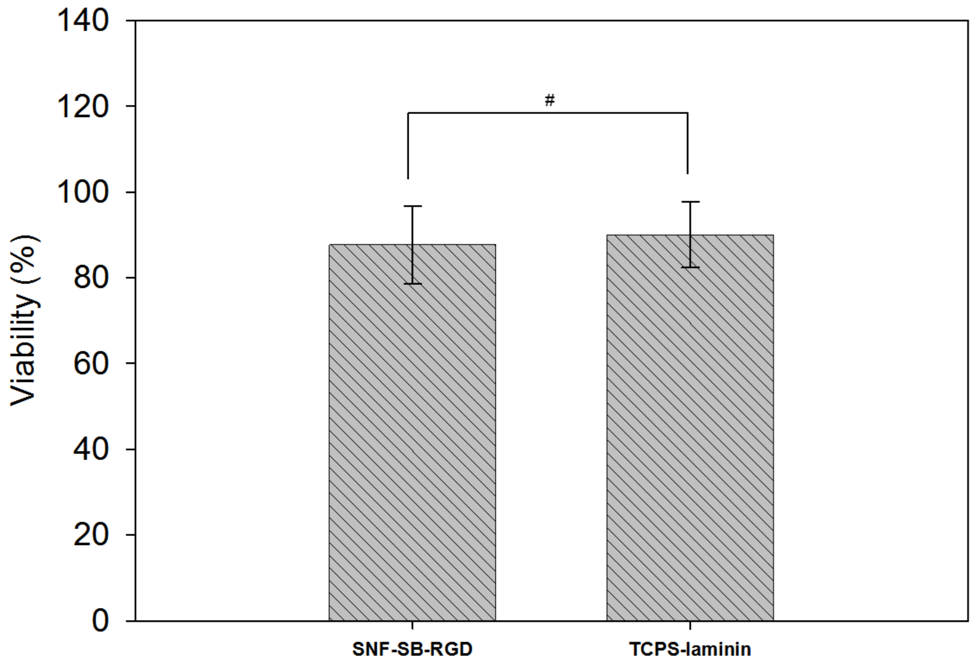

Figure. S1 Cell viability comparison between PC12 cells cultured on SNF-SB-RGD and conventional control, laminin-coated tissue culture polystyrene (TCPS-laminin), which served as the positive control. Cell viability on SNF-SB-RGD was 87.63 ± 9.02% and on TCPS-laminin 90 ± 7.68%. Assessments were done using LIVE/DEAD® stain with Ethidium Homodimer-1 and Calcein AM 72 hours after seeding. n = 10 t-test, #p >0.05.
